# Fermentation With *Pleurotus Ostreatus* Enhances the Prebiotic Properties of Germinated Riceberry Rice

**DOI:** 10.3389/fnut.2022.839145

**Published:** 2022-04-12

**Authors:** Kanjana Soodpakdee, Jutamat Nacha, Nattapol Rattanachart, Amorn Owatworakit, Sunita Chamyuang

**Affiliations:** ^1^School of Science, Mae Fah Luang University, Chiang Rai, Thailand; ^2^Microbial Products and Innovation Research Group, Mae Fah Luang University, Chiang Rai, Thailand

**Keywords:** germinated rice, *Pleurotus ostreatus*, prebiotics, β-Glucan, Riceberry rice, functional food

## Abstract

Rice is the staple food for more than half of the world's population. In recent years, awareness of the health benefits of colored rice varieties and germinated rice has gradually increased. Riceberry rice (R), a black-purple variety, was germinated and subsequently fermented with *Pleurotus ostreatus* mycelium (M) to improve nutrient quality and prebiotic properties. The γ-aminobutyric acid (GABA) and β-glucan contents were measured daily for a total of 4 days. The prebiotic activities of R, germinated Riceberry rice (GR), and germinated Riceberry rice with mycelium (GRM) were evaluated on the probiotic bacteria *Pediococcus* sp., *Lactobacillus acidophilus*, and *Streptococcus lactis*. Results were compared with the M treatment and with the commercial prebiotic agents: inulin and β-glucan. The treatments were also used to evaluate growth of the pathogen *Escherichia coli*. The GABA content peaked after 3 days of germination. The GR sample fermented with M for 3 days had the optimal concentration of both β-glucan and GABA. Evaluation of the prebiotic properties of rice samples and the commercial standards (inulin and β-glucan) showed that these were enhanced on the GR and GRM treatments. Results also showed the improvement of prebiotic properties on GR as the R sample did not show any prebiotic properties in all probiotic bacteria, whereas the GR sample showed moderate prebiotic activity score of 0.40, 0.88, and 0.56 on *Pediococcus* sp., *L. acidophilus*, and *S. lactis*, respectively. Furthermore, the prebiotic activity of GR was improved when fermented with M. For further applications, the GRM could be used on rice-based products, such as rice flour, rice crackers, or other rice products to enhance nutritional value and improve digestive system health, especially in the elderly.

## Introduction

The Earth's population is aging rapidly. The World Health Organization (WHO) has predicted that the number of people over 60 years old will be increased to 1.4 billion by 2030. Of these, 60% is anticipated to encounter more than one chronic health condition, such as neurodegenerative diseases and low-grade systemic inflammation. Recent research has postulated that changes in gut microbiota composition and abundance are associated with many of these conditions in the elderly (1). These changes include a gradual reduction of microbial taxa linked to carbohydrate-based metabolism and an increase of proteolytic bacteria. Diet is one of the primary modulatory factors of gut mirobiota. In recent years, public awareness with regard to health and food preferences has been changing. Specifically, consumption of functional foods, which enhance overall health and promote gut health, is a popular choice. Synbiotics, probiotics, and prebiotics are considered functional foods, whose beneficial effects are increasingly being revealed.

Prebiotics are non-digestible fiber compounds, which provide benefits to digestive health. In particular, prebiotics stimulate growth of probiotic, gut friendly bacteria (2). The bacterial genera that are commonly recognized as probiotics include *Lactobacillus, Bifidobacterium, Bacillus, Propionibacterium*, and *Streptococcus* (3). Recently, prebiotics have become important in the food industry because they play important roles in improving and balancing composition of gut microbiota, which in turn positively affect the host health condition (4). Common carbohydrates that function as prebiotics have been used in the human diet and include lactulose, galactooligosaccharides (GOS), fructooligosaccharides (FOS), trans-galactooligosaccharides (TOS), inulin, and β-glucan (5). Rice, especially non-white rice, is considered a good source of prebiotic substances.

In Thailand, there are several types of colored rice varieties, which have prebiotic potential. Thai black-purple rice or “Riceberry rice” (R) (*Oryza sativa* L.) is one of the most widely consumed varieties. Aside from its high nutritional and fiber content, R has potent biological properties, such as antioxidant, anti-inflammatory, anticancer, antihyperlipidemic, and hypoglycemic activities (6). Germination can enhance and improve nutrient quality of rice and other cereals. This includes increased protein, amino acid, sugar and vitamin content, and bioactive compounds such as the total amount of phenolics, γ-oryzanol, antioxidants, and γ-aminobutyric acid (GABA) (7, 8). The latter is a four-carbon amino acid neurotransmitter with anti-anxiety effects and is commonly found in colored rice varieties (9). Several studies have focused on exploring ways to increase the nutritional value of rice products. These include production of yogurt from germinated black rice and development of a prebiotic GABA drink from brown rice fermented with *Lactobacillus pentosus* 9D3, both of which have shown promise in preventing chronic diseases (10–12). Using an animal model, Lin et al. (13) showed that the **synbiotic made from** combining germinated brown rice (prebiotic) with the probiotic bacteria *Lactobacillus acidophilus* and *Bifidobacterium animalis* subsp. *lactis* could inhibit colorectal carcinogenesis by enhancing antioxidative properties and inducing apoptosis.

Germinated rice is a source of resistant starch, but its prebiotic properties have not been evaluated directly. Sawangwan and Saman (14) demonstrated the prebiotic properties of rice fermented with *Aspergillus oryzae* (solid-state fermentation). The mycelium of edible mushrooms, such as *Pleurotus ostreatus, Lentinus edodes, Auricularia auricula-judae*, and *Ganoderma lucidum*, also **constitutes a** good source of prebiotic substances, as it contains high amounts of short-chain polysaccharides, especially β-glucan (15). However, in that study, the fungal enzyme digested the rice, so that the latter could not be used further. In this study, we undertook fermentation of germinated Riceberry rice (GR) using the basidiomycete *P. ostreatus* in order to increase the prebiotic properties of the rice for further applications. We also compared the properties of the fermented germinated rice to those of non-germinated (*Oryza sativa* L.) and GR. The functional ingredients GABA and β-glucan of these three rice treatments were also measured and compared.

## Materials and Methods

### Materials

Riceberry rice (*Oryza sativa* L.) was purchased from the Chiang Rai farmer group in Chiang Rai province, Thailand. *P. ostreatus* mycelium (M) was kindly supplied by a mushroom growing farm in Chiang Rai province, Thailand. Laboratory grade chicory inulin and analytical grade GABA were purchased from Sigma-Aldrich Pte. Ltd., Singapore, and food grade β-glucan was purchased from Asia Star Trade Co., Ltd. All cultivation media and supplements were purchased from HiMedia Laboratories, LLC.

### Samples Preparation for Measuring Prebiotic Activities

In this study, R variety and *P. ostreatus* mushroom were used. The treatments were as follows: non-germinated R, GR, germinated Riceberry rice with mycelium (GRM) of *P. ostreatus*, and M. All experiments were performed in triplicate.

Germination was carried out for 48 h. The GR treatment was prepared by modifying the protocol of Chuenprasert et al. (16). Briefly, R was rinsed and soaked with sterile distilled water in plastic baskets covered by a colander for 12 h. Water was poured out, and rice was soaked again. These steps were repeated for four cycles. Ten grams of rice were collected every 4 for 48 h and processed for GABA analysis as follows: rice was dried at 60°C for 24 h and ground into flour using a grinder. The flour was then run through a sieve using a 45-μm nylon mesh to achieve equal particle size distribution. The sampling point at which the rice had the highest GABA content (at 36 h, also see below) was used for subsequent solid-state fermentation with cultivated M.

*Pleurotus ostreatus* mycelium was prepared by following the protocol of Zhu et al. (17). Briefly, M was cultivated on potato dextrose agar (PDA) for 15 days and then the mycelium on agar plate was aseptically transferred into 300 ml of potato dextrose broth (PDB). Flasks were shaken on a reciprocal shaker at 125 rpm at 25°C in darkness for 8 days after which the mycelium was harvested. A small part of the harvested mycelium was set aside for GABA analysis (see below). The rest of the harvested mycelium was used to generate the GRM treatment (in triplicate) by making a 10% mycelium inoculum of the GR total wet weight and performing solid-state fermentation. The fermentation process was carried out for 4 days in static condition at 25°C in darkness. During the fermentation experiment, 10 g of GRM sample was collected daily for GABA and β-glucan analysis. Sample was processed into flour as described above.

To measure the prebiotic properties of the treatments (R, GR, GRM, and M), samples were collected and processed into flour as described above. For this purpose, the GR sample was collected at 36 h during germination, GRM at 72 h during fermentation, and M at 72 h during cultivation in PDB.

### Bacterial Strains

Three Gram-positive lactic acid bacteria, such as *Pediococcus* sp. (strain TISTR 129)*, L. acidophilus* (strain TISTR 2365), and *Streptococcus lactis* (strain TISTR 457), and the Gram-negative bacterium *Escherichia coli* (strain TISTR 527) were used in this experiment. Lactic acid bacteria were inoculated in de Man Rogosa Sharpe (MRS) broth medium (Himedia) according to Rokana et al. (18). *E. coli* was maintained on nutrient broth (NB) from Himedia and incubated at 37°C for 16–24 h.

### Analysis of the GABA Content in Rice Samples

The extraction procedure was modified from Gökmen et al. (19). One gram of each rice flour sample and ground mycelium sample was extracted using deionized (DI) water in a ratio of 1:15 w/v. The suspension was incubated in a water bath (Memmert WNE-22) at 90°C for 5 min. The supernatant was diluted with 50% (v/v) of acetonitrile and filtrated through a 0.45-μm nylon filter prior to analysis using Liquid Chromatography Mass Spectrometry Triple Quadrupole.

The GABA content of the rice and mycelium extracts used in this study were analyzed based on the method previously described by Qiu et al. (20). In brief, the GABA standard was prepared in a range of 0–100 ppm to construct a standard curve. The GABA standard and extracted samples were analyzed with the Liquid Chromatography Mass Spectrometry Triple Quadrupole system (Shimadzu, Nexera X2/LCMS 8060) using an Infinity Lab Poroshell 120 HILIC-Z column (2.1 × 100 mm, 2.7 μm). Injection volume was 1 μl, and the flow rate was set at 400 μl/min at 30°C. A linear gradient system was used with mobile phase A (0.1% formic acid in DI water) and mobile phase B (10 mM ammonium formate in acetonitrile) as follows: 6% solvent B for 3 min (0–3 min), 27% B for 4 min (3–7 min), 27% B held for 1 min, then to 37% B for 1 min (8–9 min), and back to 0% B for 1.5 min (9–10.5 min). The column was re-equilibrated for 8.5 min (10.5–19 min) under the initial conditions (0% B). Total run time was 19 min including column re-equilibration.

### Beta Glucan Assay

The β-Glucan Assay Kit Yeast and Mushroom (Megazyme, Ltd.) was used to determine content of 1,3- and 1,3/1,6-β-D-glucans in rice and mycelium extracts in this study. The assay method was according to the standard protocol as stated by McCleary and Draga (21). Determination of β-glucan content was performed in duplicate as suggested by the manufacturer's instructions (version 02/17).

### Growth Stimulation of Probiotic Bacteria

The basal medium for cultivation of bacteria contained 1% lactose, 0.1% peptone, and 0.1% yeast extract medium and was used as a lactose control medium. For the test experiments, lactose was replaced with 1% of sample (ground form for R, GR, GRM, and M; and powder form for inulin and β-glucan). Hence, each bacterial strain was grown in seven separate tubes containing: 1% lactose, 1% R, 1% GR, 1% GRM, 1% M, 1% inulin, and 1% β-glucan. The process was done in triplicate for each bacterial strain. Bacterial suspensions were prepared at a concentration of 0.05 at OD_600_ and 1% v/v of each bacterial suspension was inoculated in separate media. The inocula were incubated at 37°C, while shaking at 200 rpm for 24 h. Each treatment was sampled every 4 h for a total of 24 h to determine growth based on the number of colony-forming units (CFUs).

### Prebiotic Activity Score

The performance of the experimental treatments was evaluated using the prebiotic activity score (P_A_) according to Figueroa-Gonzalez et al. (22). The score was based on whether substrates supported growth of probiotic strains and compared with that of other organisms, in this case with *E. coli* (strain TISTR 527). Prebiotic activity score was calculated by using cell count (CFU/ml) as follows:


$$\begin{array}{l} P_{A}=\;\frac{\left(LogP24-LogP0\right)pre}{\left(LogP24-LogP0\right)lac}-\frac{\left(LogE24-LogE0\right)pre}{\left(LogE24-LogE0\right)lac} \end{array}$$

where, P_A_ is the prebiotic activity score. Log P is the log of growth of probiotic bacteria (CFU/ml) cultured on lactose (Lac) and prebiotic supplements (Pre) at 0 h (P_0_) to 24 h (P_24_), Log E is log of growth of growth (CFU/ml) of *E. coli* cultured on lactose (Lac) and prebiotic supplements (Pre) at 0 h (E_0_) to 24 h (P_24_).

If the P_A_ is lower than 1, it means that the growth of the tested strain is lower on a specific prebiotic compared to the control carbohydrate, and/or its growth is lower than the reference bacteria (*E. coli*). On the contrary, if the substrate has a high prebiotic activity score, it will support the growth of probiotic bacteria (23).

### Dinitrosalicylic Acid Method

Media samples (from Section Growth Stimulation of Probiotic Bacteria) were taken from each treatment at 0 and 24 h to determine the total amount of reducing sugars left in the media by using the dinitrosalicylic acid method. This method was modified from Başkan et al. (24). Briefly, 3–5-dinitrosalicylic acid (DNS) was dissolved in 100 ml of 2 M NaOH and KC_4_H_5_O_6_ was added and adjusted to 500 ml with water. One ml of DNS was mixed with 1 ml of sample using a vortex and incubated in a water bath at 95°C for 5 min. The mixture was then cooled down in an ice bath, and 3 ml of water was added and mixed. The absorbance of samples was measured at 540 nm. A glucose standard curve was established using known concentrations of glucose. Concentrations of glucose in the treatments were determined using linear regression.

### Statistical Analysis

Data analysis was performed using IBM SPSS Statistics Program Version 23 (SPSS, Inc., Chicago, IL, USA) using ANOVA, followed by the Duncan multiple comparison test. A *p-*value of < 0.05 denoted statistical significance. All experiments were done in triplicate, and results are expressed as mean values with standard deviations.

## Results and Discussion

### LCMS-MS-MS for GABA Analysis

**Figure 1:**
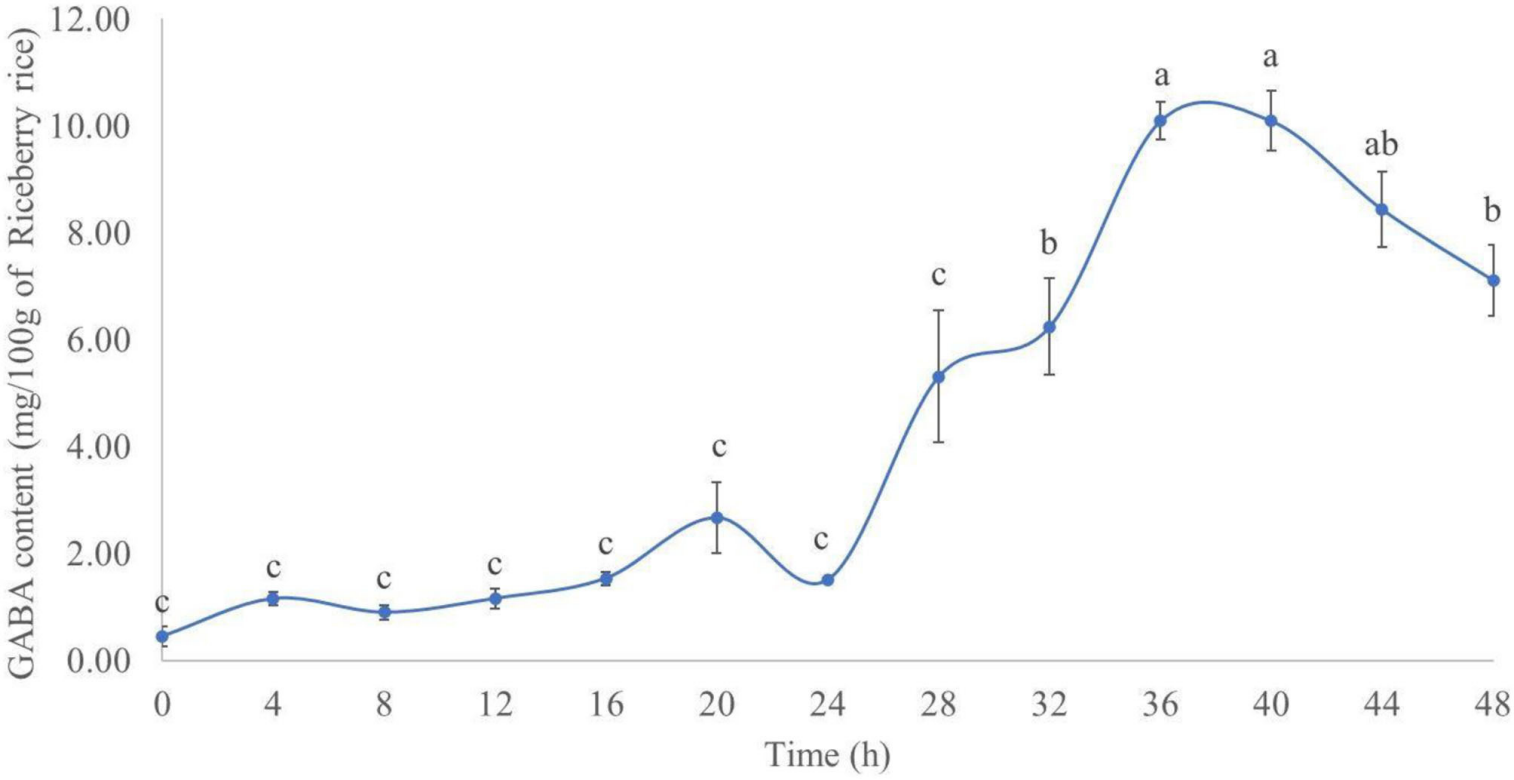
GABA content (mg/100 g of dry Riceberry rice) in germinated rice between 0 and 48 h. Results with the same superscript were not significantly different (*p* > 0.05).

The results of the GABA content during rice germination are presented in Figure 1. In the R, the GABA content increased for up to 40 h after germination. The highest GABA content (mg/100 g of dried R) was 10.10 ± 0.36 at 36 h after germination, followed by 10.09 ± 0.56 at 40 h. The difference between the GABA content of the two time points was not statistically significant (*p* > 0.05). The experimental results herein agree with previous findings by Tumpanuvatr et al. (25) and Wang et al. (26), both of them showed that the GABA content increased during the rice germination process. This could be explained by the activation of the glutamate decarboxylase (GAD) enzyme and subsequent formation of GABA from L-glutamic acid, hence increasing the GABA content in germinated rice (25, 26). Based on these results, rice germinated for 36 h was used for further experiments, due to the highest GABA content and shorter time.

### GABA and β-Glucan Determination in Germinated Rice With Mycelium Sample

**Figure 2:**
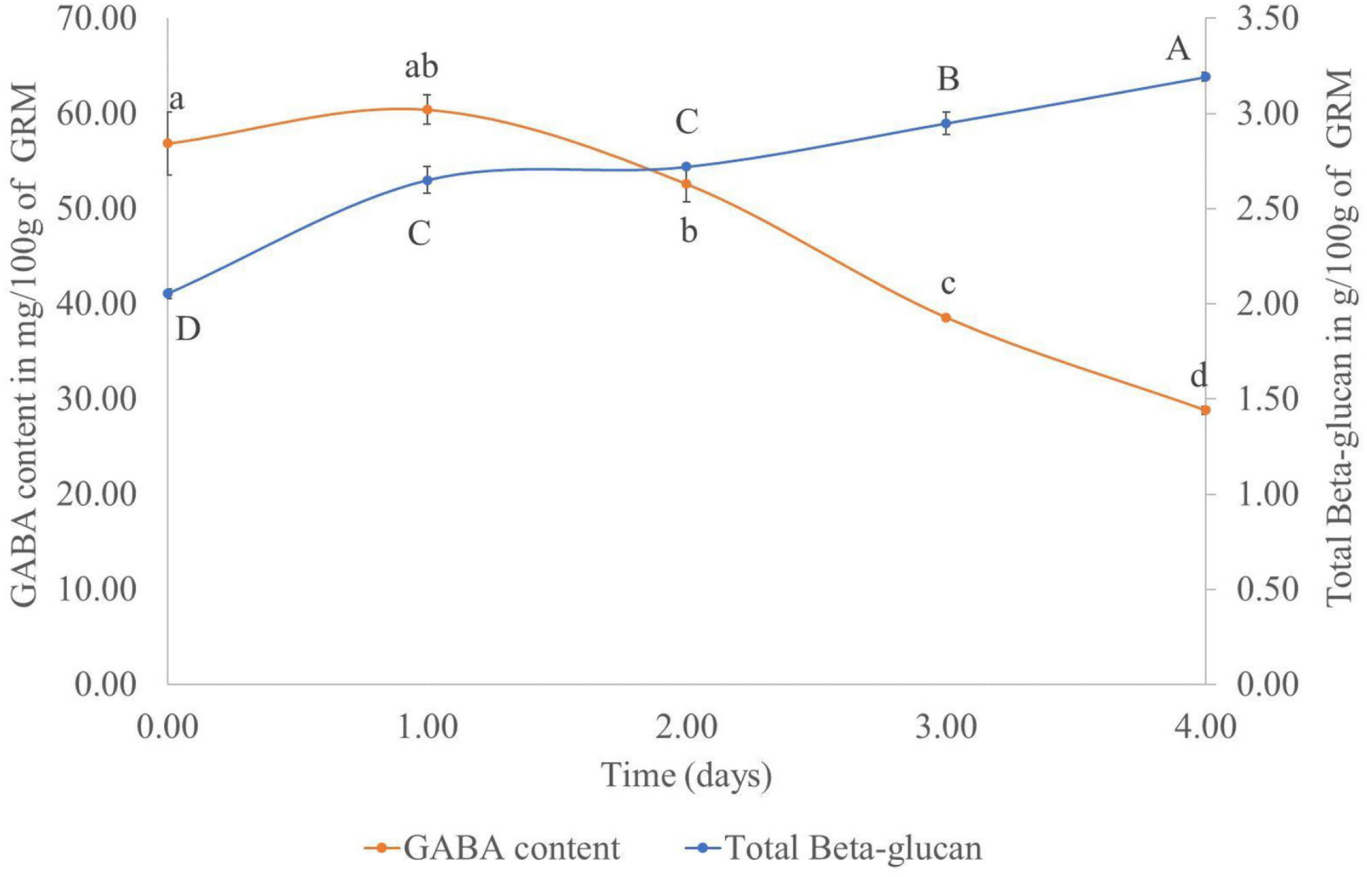
Total β-glucan and GABA contents (mg/100 g sample) in germinated rice with mycelium of *Pleurotus ostreatus* from day 0 to 4. Points with the same superscript on the same line were not significantly different (*p* > 0.05).

To determine the optimal amount of time for cultivating *P. ostreatus* on the GR, the GABA and β-glucan contents of the cultivated rice samples was measured daily for 4 days. Results showed that in the GRM sample, the GABA content decreased, whereas the β-glucan content showed an increasing trend (Figure 2). Based on the results, the day 2 GRM sample was not chosen, as the M did not fully cover the rice at that time. The GRM sample at day 3 was selected for further experiments. The day 4 GRM sample was excluded, as the GABA content was dramatically reduced, with a reduction of 49.30% from day 1 to 4. The β-glucan content was progressively increased reaching 2.95 ± 0.06/100 g dried sample on day 3, which was close to the β-glucan content on day 4 (3.19 ± 0.02/100 g dried sample). Thus, the day 3 GRM sample was deemed suitable to use for prebiotic properties experiment.

Notably, the GABA content in GR cultivated with M did not decrease from day 0 to 1. This might be due to the lag stage of the growth of mushroom mycelium. The dramatic decrease of the GABA content from day 1 to 4 of solid-state fermentation could be due to the enzymatic breakdown of complex molecules, such as protein, amino acid, starch, lignocelluloses, and hemicellulose, by the fungal enzymes. The broken down products could be used to sustain growth of the mycelium (27). This finding was in contrast with previous studies by Cai et al. (28) and Wan-Mohtar et al. (29). These investigators used *A. oryzae* to improve the GABA content in oats and soybeans koji, respectively. The noted increase in the GABA content of grains after fermentation with *A. oryzae* could be explained by the decarboxylation of glutamic acid by GAD of *A. oryzae* (28). GAD is the rate-limiting enzyme of GABA synthesis. Furthermore, Wan-Mohtar et al. (29) reported that the carbon-to-nitrogen ratio also had an effect on GABA production with the ratio C8:N3 being the best in koji fermentation with *A. oryzae*. In this study, we focused on the GABA content in GR (Figure 1) rather than its production by *P. ostreatus*. Nevertheless, the GABA content in *P. ostreatus* samples was also measured. On day 3, the mycelium treatment contained 1.15 ± 0.14 mg GABA per 100 g of dried mycelium. The GABA content increased to 23.08 ± 2.83 mg per 100 g dried mycelium after cultivation of the fungus for 60 days using submerged fermentation, whereas the fruiting body of *P. ostreatus* contained 31.74 ± 1.58 mg GABA per 100 g dried sample. This finding also showed that the GABA content could be increased with the cultivation of *P. ostreatus* mycelium. However, the production time is too long, and by that time, the rice might be broken down by the mushroom mycelium meaning that it could not be used further for rice flour production.

Contrary to GABA, the β-glucan content increased with time. The increase could be attributed to the growth of the mushroom mycelium, as β-glucan is one of the fungal cell wall components (30).

### Probiotic Growth Stimulation

The cell densities of *Pediococcus* sp., *L. acidophilus, S. Lactis*, and *E. coli* were increased following 24 h of growth on media containing 1% (w/v) of prebiotic supplements (Table 1). *Pediococcus* sp. and *L. acidophilus* had the highest growth rate in the commercial β-glucan media, whereas inulin promoted the growth of *Pediococcus* sp. and *S. lactis*. The growth rate of *S. lactis* was highest in GRM than in the rest of the treatments. Even though GR promoted the growth of probiotic bacteria similarly to the GRM sample, the former also promoted the growth of *E. coli*, whereas this was not observed in the latter. Based on the results of this experiment, germination seemed to increase the prebiotic properties of rice, whereas fermentation with the mushroom mycelium further improved these properties in the germinated rice. This might be due to the GRM sample containing β-glucans from *P. ostreatus*. This finding is in agreement with those of Cheng et al. (31) and Chaikliang et al. (32). Even though rice contains carbohydrates, such as starch and fiber, these could not serve as prebiotic agents. Enzymatic breakdown using a maltogenic amylase, such as bacterial β-amylase, could produce maltodextrin and glucose syrup, which may promote growth of pathogenic bacteria such as *E. coli* (33). Furthermore, previous studies showed growth of *E. coli* not only on prebiotics but also on media with minimal amount of carbohydrates. The latter could be explained by presence of alternative metabolic pathways that enable the bacterium to breakdown nutrients independent of glucose fermentation (34, 35). Nevertheless, when probiotic bacteria were cultured along with *E. coli* with supplementation of prebiotics, the pH of the culture medium was decreased due to the acid production by probiotic bacteria, which in turn prevented growth of *E. coli* (34). The results of this study clearly showed that *E. coli* could utilize the sugar in rice more easily than the complex polysaccharides in β-glucans from M. In support of this, the total reducing sugar content after 24 h of culturing bacteria was decreased in all samples (Table 2).

**Table 1:** Cell density at 0 and 24 h, reported as Log CFU/ml, for bacterial cultures grown in different treatments.

**Bacteria**		**Log of growth prebiotic supplements (CFU/ml)**							
		**Lac**	**In**	**Neg**	**R**	**GR**	**M**	**GRM**	**β-glucan**
PE	0 h	7.12 ± 0.03^i^	7.22 ± 0.02^i^	7.44 ± 0.05^h^	7.19 ± 0.01^i^	7.12 ± 0.12^i^	7.60 ± 0.09^g^	7.22 ± 0.07^i^	7.65 ± 0.04^g^
	24 h	10.50 ± 0.09^b^	10.62 ± 0.02^a^	8.09 ± 0.10^f^	9.39 ± 0.10^e^	9.84 ± 0.04^c^	9.58 ± 0.15^d^	9.92 ± 0.04^c^	10.70 ± 0.03^a^
LA	0 h	6.12 ± 0.06^j^	7.07 ± 0.04^h^	6.51 ± 0.10^i^	6.44 ± 0.02^i^	6.74 ± 0.04^i^	7.00 ± 0.09^h^	6.32 ± 0.01^i^	7.02 ± 0.01^h^
	24 h	12.05 ± 0.00^d^	12.39 ± 0.03^c^	8.43 ± 0.04^f^	8.20 ± 0.01^g^	6.39 ± 0.03^i^	12.89 ± 0.07^b^	9.97 ± 0.03^e^	12.99 ± 0.06^a^
SL	0 h	7.44 ± 0.02^i^	7.25 ± 0.01^j^	6.92 ± 0.06^k^	7.50 ± 0.09^i^	6.76 ± 0.07^k^	6.28 ± 0.09^l^	6.77 ± 0.09^k^	6.27 ± 0.04^l^
	24 h	11.57 ± 0.03^b^	12.20 ± 0.00^a^	9.66 ± 0.10^Ce^	8.88 ± 0.01^g^	7.84 ± 0.03^h^	9.56 ± 0.09^f^	10.22 ± 0.02^c^	10.01 ± 0.04^d^
EC	0h	5.92 ± 0.08^i^	5.94 ± 0.09^i^	5.86 ± 0.09^j^	5.92 ± 0.09^i^	5.73 ± 0.06^k^	6.21 ± 0.02^g^	6.14 ± 0.02^g^	6.09 ± 0.02^h^
	24h	12.21 ± 0.05^c^	12.01 ± 0.04^d^	11.92 ± 0.04^d^	13.36 ± 0.07^a^	12.58 ± 0.03^b^	11.70 ± 0.06^e^	11.77 ± 0.02^e^	11.26 ± 0.10^f^

*PE, LA, SL, and EC refer to Pediococcus sp., Lactobacillus acidophilus, Streptococcus lactis, and Escherichia coli respectively. Lac, In, Neg, R, GR, M, and GRM refer to lactose, inulin, negative media (without sugar), Riceberry rice, germinated Riceberry rice, P. ostreatus mycelium, and germinated Riceberry rice with mycelium, respectively. Each bacterial strain is analyzed independently from other strains. The different superscript letters (a–l) indicate significant differences (p < 0.05)*.

**Table 2:** Total reducing sugar content in sample used in this study.

**Bacteria**		**Total reducing sugar in prebiotic supplements (mg/ml)**							
		**Lac**	**In**	**Neg**	**R**	**GR**	**M**	**GRM**	**β-glucan**
PE	0 h	7.18 ± 0.03^a^	0.64 ± 0.02^e^	0.25 ± 0.06^f^	0.56 ± 0.05^e^	1.00 ± 0.00^d^	0.90 ± 0.02^d^	0.84 ± 0.05^d^	1.95 ± 0.01^c^
	24 h	6.54 ± 0.01^b^	0.54 ± 0.01^d^	0.12 ± 0.03^f^	0.45 ± 0.02^e^	0.83 ± 0.03^d^	0.47 ± 0.03^e^	0.61 ± 0.03^e^	0.61 ± 0.01^e^
LA	0 h	8.07 ± 0.02^a^	0.86 ± 0.01^d^	0.23 ± 0.00^h^	0.51 ± 0.01^f^	0.0.73 ± 0.01^e^	0.71 ± 0.01^e^	0.91 ± 0.00^d^	2.18 ± 0.01^c^
	24 h	5.00 ± 0.01^b^	0.44 ± 0.01^f^	0.07 ± 0.01^i^	0.34 ± 0.03^g^	0.38 ± 0.01^g^	0.30 ± 0.01^g^	0.46 ± 0.00^f^	0.47 ± 0.00^f^
SL	0 h	7.56 ± 0.01^a^	0.72 ± 0.03^f^	0.23 ± 0.00^i^	0.57 ± 0.03^g^	1.08 ± 0.01^e^	0.70 ± 0.03^f^	1.38 ± 0.02^d^	2.09 ± 0.02^c^
	24 h	5.41 ± 0.01^b^	0.19 ± 0.00^i^	0.08 ± 0.00^j^	0.43 ± 0.02^g^	0.66 ± 0.00^g^	0.34 ± 0.01^h^	0.33 ± 0.01^h^	0.75 ± 0.01^f^
EC	0 h	6.19 ± 0.01^a^	0.63 ± 0.00^g^	0.28 ± 0.03^j^	0.51 ± 0.01^h^	1.00 ± 0.00^e^	1.18 ± 0.02^d^	1.07 ± 0.00^e^	2.17 ± 0.02^c^
	24 h	3.65 ± 0.01^b^	0.34 ± 0.00^i^	0.14 ± 0.03^k^	0.27 ± 0.03^j^	0.77 ± 0.01^f^	0.38 ± 0.01^i^	0.41 ± 0.00^i^	0.52 ± 0.00^h^

*PE, LA, SL and EC refer to Pediococcus sp., Lactobacillus acidophilus, Streptococcus lactis, and Escherichia coli respectively. Each bacterial strain is analyzed independently from other strains. The different superscript letters (a–k) indicate significant differences (p < 0.05)*.

### Prebiotic Activity Score

**Figure 3:**
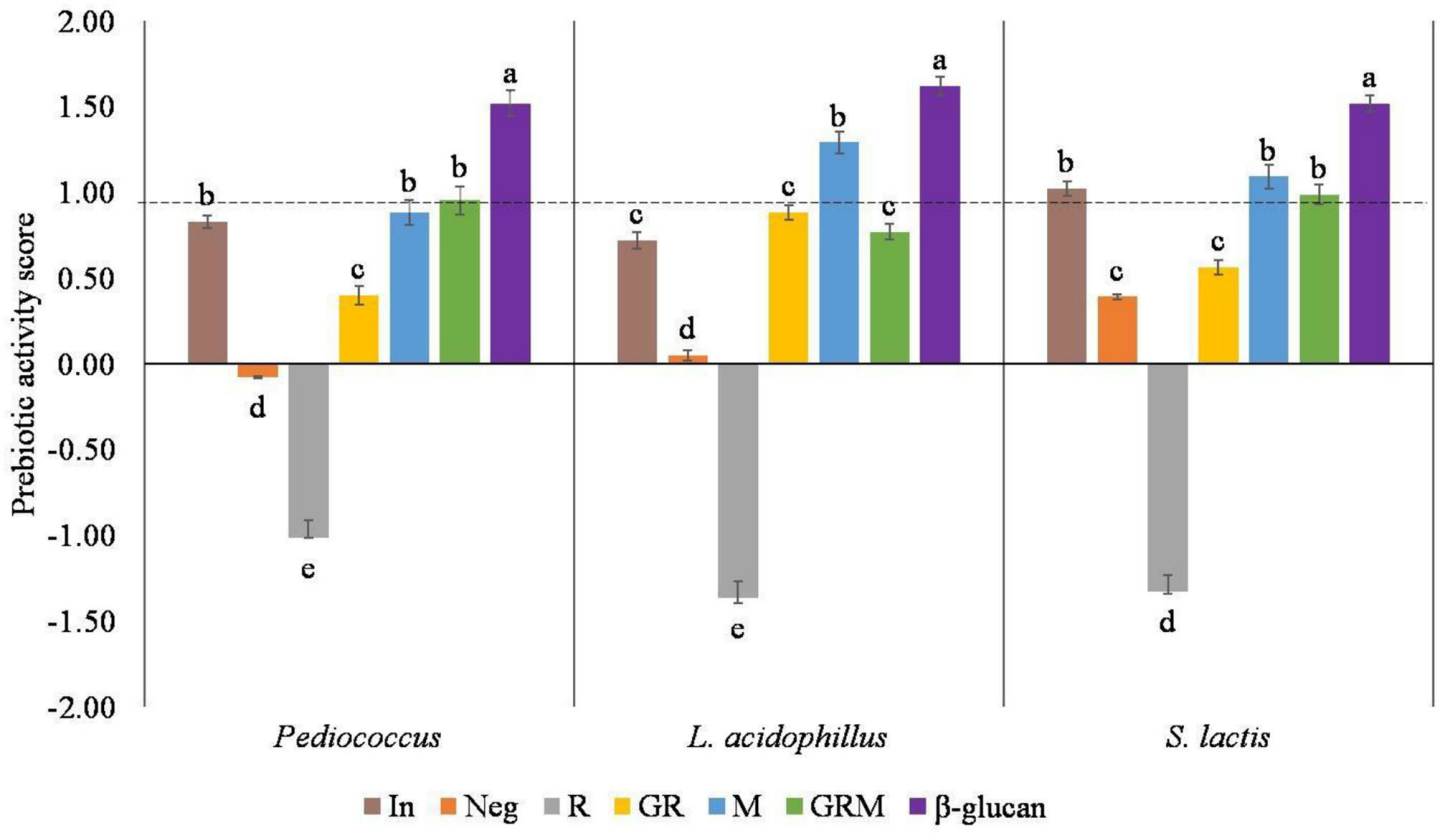
Prebiotic activity score in different samples. In, Neg, R, GR, M, GRM, and β-glucan: inulin, negative control, Riceberry rice, germinated rice, oyster mushroom mycelium, and mushroom, germinated rice berry with oyster mushroom mycelium and β-glucan. Results in the same strain of bacteria with the same superscript were not significantly (*p* > 0.05) different.

Prebiotic activity score has been described as the ability of a given prebiotic substrate to promote the growth of probiotic bacteria compared with other organisms. In this study, the probiotic bacterium *Pediococcus* sp., *L. acidophilus*, and *S. lactis* were used and compared with *E. coli*. A higher score reflects a higher prebiotic activity, whereas a low or negative value of prebiotic activity score indicates that probiotic bacteria favor utilization of the prebiotics medium to a lesser degree when compared to lactose utilization at the same fermentation time (36). Based on the prebiotic activity scores shown in Figure 3, the R treatment had no prebiotic activities toward these three probiotics bacteria. Germination improved prebiotic properties of rice samples, but not to the level of inulin, the commercial prebiotic standard. Despite the highest prebiotic activity score of β-glucan, the prebiotic properties of germinated rice were improved by cultivation with M for 3 days. The prebiotic activities in *Pediococcus* sp. and *S. lactis* were not significantly different than those observed in inulin, M, and GRM.

The improvement of prebiotic activities in germinated rice could be due to the enzymatic breakdown of carbohydrates into oligosaccharides, which may have acted as prebiotic agents (37). Furthermore, the prebiotic activities of GRM could come from the combination of germinated rice and β-glucan in M. In this study, an increased GABA content was achieved by germination, whereas prebiotic properties in R were enhanced with cultivation with M. The derived rice sample could be used further in rice-based products.

## Conclusions

The nutritional and prebiotic properties of R could be enhanced by germination and fermentation with mushroom mycelium. GR showed a higher GABA content, and its prebiotic properties were better than those of non-germinated rice. In this study, the GRM showed a comparable GABA content with GR and improved prebiotic properties toward *Pediococcus* sp. and *S. lactis*. The prebiotic activity score of GRM did not differ significantly from that of inulin, which is widely used as a prebiotic agent in the food and supplement industry. Thus, the flour from GRM could be used on rice-based products, such as rice flour, rice crackers, or other rice products to enhance nutritional value and improve digestive system health, especially in the elderly. In the future, the scope of the study can be expanded to using GR and GRM for anti-colorectal cancer (CRC) and anti-Alzheimer's disease as both treatments contain GABA, β-glucan, and other non-digestible polysaccharides.

## Data Availability Statement

The raw data supporting the conclusions of this article will be made available by the authors, without undue reservation.

## Author Contributions

SC: conceptualization, writing—review, editing, and funding acquisition. AO: methodology. AO and SC: validation and supervision. KS, JN, and NR: formal analysis. KS and JN: investigation and writing—original draft preparation. JN: project administration. All authors have read and agreed to the published version of the manuscript.

## Funding

This research was funded by the Thailand Science Research and Innovation (TSRI) with Grant Number 642B01002 awarded to SC.

## Conflict of Interest

The authors declare that the research was conducted in the absence of any commercial or financial relationships that could be construed as a potential conflict of interest.

## Publisher's Note

All claims expressed in this article are solely those of the authors and do not necessarily represent those of their affiliated organizations, or those of the publisher, the editors and the reviewers. Any product that may be evaluated in this article, or claim that may be made by its manufacturer, is not guaranteed or endorsed by the publisher.
